# News: Regions

**Published:** 2011-12

## Africa

### Cape Verde's Pedro Verona Pires awarded Mo Ibrahim African leadership prize

Cape Verde's former president Pedro Verona Pires won the US$ 5 million (€ 3.8 million) “African leadership prize” awarded by the Mo Ibrahim Foundation. The prize was created in 2007, it is worth US$ 5 million (€ 3.8 million) over 10 years and then US$ 200 000 (€ 154 333) annually for life thereafter. Previous winners included Mozambique's former president Joaquim Chissano and Botswana’s Festus Gontebanye Mogae, while Nelson Mandela of South Africa was named an honorary laureate in 2007. This year the prize was given to Mr Pires for transforming his small country into a model of democracy and stability and then deciding to step down after his second term. Speaking for The Guardian, Salim Ahmed Salim, the chair of the prize committee, has said that “...under 10 years as president, the nation became only the second African country to graduate from the United Nation's least-developed category and has won international recognition for its record on human rights and good governance”. Pires was appointed first prime minister of independent Cape Verde in 1975, and he remained in this post for 16 years. He lost his country's first democratic elections in 1991, but was elected again in 2001 and then re-elected in 2006. For the past two years the Foundation, set up by a Sudan-born billionaire Mo Ibrahim, did not award a prize as no leaders met the criteria for promoting development and democracy and for handing over power peacefully.

### Vaccine introduction reduced pneumonia infections in Kenya by more than two-thirds

In a recent study, conducted by the Kenya Medical Research Institute Wellcome Trust Research Programme, pneumonia infections in Kilifi, Kenya, have dropped by 70% since the introduction of the pneumococcal vaccine. Earlier this year, in February 2011, Kenyan president Mwai Kibaki made the pneumococcal vaccine available to all Kenyan children. The vaccine is now freely available in about 3200 health facilities across Kenya that offer immunization. The vaccine targets infants in their first year of life and is given in three doses. Beth Mugo, a minister for public health, urged mothers to ensure that their children have the course completed to prevent resistance to the vaccine. This is of particular importance because early data showed that more than 20% of the vaccinated children did not complete the full course. Annually, the government will be contributing 72 million Kenyan shillings for the vaccine introduction. As the vaccine is now available throughout the country, the local experts believe that the results of the study are indicative for the country as a whole.

### H3Africa aims to bring research capacity in genomics to the poorest continent

H3Africa stands for the “Human Heredity and Health in Africa”. This welcome initiative aims to facilitate a contemporary research approach to genetic and environmental determinants of common diseases, resulting in an improved health among African populations. This should be achieved through the development of the necessary expertise among African scientists, and also establishing networks of African investigators. Current genomic research in wealthy countries is focused on developing tools for early and more accurate diagnosis, the development of new drugs and, potentially, personalized medicine – which has become a term describing the idea of systematic use of information about each individual to select or optimize the care provided to each patient individually. However, most African countries are being left out of the ongoing genomic revolution. The sponsors behind this initiative are the African Society of Human Genetics, the National Institutes of Health in the USA and The Wellcome Trust in the UK.

### BMJ's publication on the cost of Africa's doctor brain drain appears flawed

In 2010, the World Health Assembly adopted the first “Code of Practice on the International Recruitment of Health Personnel”. The idea behind this code was to recognise problems associated with doctor migration, implications on health systems in low resource settings, and to call on wealthy countries to provide financial assistance to source countries affected by health worker losses. In late November this year, a study published by BMJ claimed Sub-Saharan African countries that invest in training doctors “*have ended up losing US$ 2 billion as the expert clinicians leave home to find work in more prosperous developed nations*”, as Reuters subsequently reported. But only weeks later Michael Clemens from the Center for Global Development ridiculed the simplistic approach to that calculation, providing six important arguments that all relate to the complexities of assessing the costs and benefits of highly skilled workers to their home nation – none of which had been considered in the original article, and all of which would probably entirely change the conclusions.

### Lead poisoning epidemic in Nigerian villages

United Nations reported that more than 40 villages in Nigeria have been presenting cases of lead poisoning and called for an increase in preventive measures. The World Health Organization (WHO) has been assisting the Nigerian Government in dealing with the epidemic. WHO recommended strengthening the capacity to diagnose and treat the illness and ensuring de-contamination. WHO also warned about mining practices which are thought to be causing the sickness, with ore processing activities and storage of ore materials too close to the villages, using the obsolete practices with produce too much dust, and failure to remove contaminated clothes and wash before returning home. Lead poisoning damages the nervous system and causes brain and blood disorders, with long-term and expensive treatment with chelating agents required, which eventually remove heavy metals from the body. According to WHO, children in several villages in Zamfara state already require chelation therapy. Since the problem was discovered last year, nearly US$ 2 million (€ 1.5 million) has been provided by the UN Central Emergency Response Fund (CERF) to WHO and UNICEF to provide treatment, train doctors, provide diagnoses and raise awareness about the hazards of lead.

## Asia

### Afghanistan's largest mortality survey highlights improved maternal health

The Afghanistan Mortality Survey (AMS) was completed in 2010 as a part of the worldwide Demographic and Health Surveys (DHS) project. The survey showed a significant decrease in maternal mortality – to about 500 deaths per 100 000 live births. This is a truly significant decrease – down from 1800 per 100 000 live births in the UN’s 2005 report. The AMS report suggests the decrease could be linked to the increased levels of antenatal care received by pregnant women. In the 36-59 months preceding the survey, 57% of women received antenatal care from a skilled provider, which rose to 68% of all pregnancies over the 12 months prior to the AMS survey. However, the report concedes that there is still a lot of work to be done, especially in regards to access to health care for women. The report found that 70% of women highlighted lack of money and distance to health care facilities as major barriers to accessing antenatal care.

### MSF's outrage over CIA's fake vaccination campaign serving secret operations in Pakistan

As revealed by The Guardian, the international medical aid charity Médecins Sans Frontičres (MSF) have lashed out at the CIA for using “...a fake vaccination programme as a cover to spy on Osama bin Laden». MSF said that this episode could threaten life-saving immunisation work around the world. The international medical aid charity added that this “...ploy used by US intelligence was a grave manipulation of the medical act”. The CIA recruited a Pakistani doctor and health visitors before the operation that killed Bin Laden in Abbottabad, in northern Pakistan, to confirm that the al-Qaida leader was indeed living in the compound. The doctor set up a vaccination drive for Hepatitis B in the town, to gain entry to the Bin Laden compound and obtain DNA samples the residents. Speaking for the Guardian, a senior US government official defended the practice, saying that it had been intended as “...an actual vaccination campaign conducted by real medical professionals”. Later in the year, a team from the non-governmental organisation Save the Children needed to evacuate from its operations site in Pakistan amid safety concerns.

### 50th anniversary of universal health care in Japan

In April, Japan marked the 50th anniversary of universal health care. Since its introduction, Japan became a leading nation in several health metrics, most notably longevity. To mark this anniversary, The Lancet published a series of six theme papers and eight comments by Japanese academics. According to The Lancet, as they describe Japan's actions and provide an opportunity to translate that experience to other settings, the invited theme papers resemble “...finely crafted netsuke (fasteners): both functional and provoking reflection”.

### A study reminds of a less prominent cause of malaria

The most common form of malaria in Africa, which is caused by *Plasmodium malariae*, has been the subject of intense research interest and investments of the Global Fund and other stakeholders. However, a less dangerous form of the disease – the one caused by *Plasmodium vivax* – is still very prevalent or endemic in large parts of South Asia and Latin America. Recently, a new global map of the *P. vivax* malaria parasite has been revealed. It showed that the disease is endemic in substantial parts of the world. Kevin Baird, a researcher working with the Eijkman-Oxford clinical research unit in Indonesia, said that all the new information increasingly showed that *P. vivax* was a bigger threat than generally thought. Primaquine is the only treatment for *P. vivax*, while all the current vaccine efforts are targeting *P. falciparum*.

### Bangladesh plans to immunize 90% of children with major vaccines by 2016

Among six large populous countries eligible for GAVI support, Bangladesh earned recognition as the best performer in immunization coverage, after reducing the number of unimmunized children by more than 50% over the past four years. Bangladesh’s Prime Minister Sheikh Hasina expressed her firm commitment to achieving the Millennium Development Goals in different sectors in Bangladesh, including child health. She said that 80% of children in Bangladesh have already been brought under the immunization programs and her current plan is to increase the coverage to 90% by 2016. UN honoured Sheikh Hasina by giving her Millennium Development Goal (MDG) Award last year.

## Australia and Western Pacific

### Kevin Rudd summarizes four years of foreign policy achievements

In November this year, the federal government of Australia completed their fourth year in charge, which also included the decisions made on foreign policy. The present minister of foreign affairs, Mr Kevin Rudd, gave a speech to the Australian Institute of International Affairs, outlining the challenges faced, achievements and visions of his government’s foreign policy. The major focus of the speech was on the importance of Australia being positive, outward looking and globally engaged as a country. In his visions, he laid out 10 key goals to be focused on in Australia’s foreign policy. He mentioned how in 2006 and 2007 Australian support for the Global Alliance for Vaccines and Immunisations (GAVI) funded the vaccination of 500 000 children against disease and how his government supported the further vaccination of 1.1 million children by the end of 2010 and pledged to vaccinate further 7.7 million by 2015. Australia also committed heavily to the Global Partnership for Education, which resulted in over 2 million children being enrolled and completing primary education. Mr Rudd also gave notice to Australia having been one of the largest bilateral donors of humanitarian aid in the recent Horn of Africa crisis.

### Priority actions to tackle non-communicable disease (NCD) pandemics

In April, *The Lancet* published an important series of papers, led by Auckland-based Dr Robert Beaglehole on behalf of the Lancet's NCD Action Group and NCD Alliance. The series was published ahead of the UN's High-Level Meeting on Non-Communicable Diseases (NCDs). The authors proposed “…five overarching priority actions for the response to the crisis leadership, prevention, treatment, international cooperation, and monitoring and accountability and the delivery of five priority interventions: tobacco control, salt reduction, improved diets and physical activity, reduction in hazardous alcohol intake, and essential drugs and technologies”. The priority criteria were their health effects, cost-effectiveness, low costs of implementation, and political and financial feasibility. Tobacco control was seen as the most urgent priority, while an estimated global commitment of about US$ 9 billion (€ 7 billion) per year would be required to bring enormous benefits to social and economic development and to the health sector.

### Australia’s Prime Minister joins The Gates Foundation in fight against polio

Polio has not been diagnosed in Australia for 40 years, but three of the four countries where it is still endemic – Nigeria, Pakistan and India – are members of the Commonwealth. This is why Australia's Prime Minister Julia Gillard chose to address the polio initiative, among other topics, at the Commonwealth Heads of Government Meeting earlier this year. She also published a joint opinion piece with Bill Gates in the Fairfax papers on the final push to eradicate polio earlier this year. Helen Evans, from the GAVI in Geneva, said for the ABC’s The News Today that she expected the Commonwealth Heads of Government Meeting to pressure countries where the disease still exists to act and eradicate it. Up until October 2011, the four countries recorded 429 new cases, which is down from 706 last year, but given that it is a highly infectious disease, the world cannot relax until it is fully eradicated. Complacency could see hundreds of thousands of new cases if immunization efforts were relaxed. Ms Evans added that The Bill and Melinda Gates Foundation have already put a huge amount of money into polio and that they were now “… calling on governments to join with them for this last piece of the fight”.

### Australian health official managed to misappropriate US$ 16 million intended for charities

According to Sydney’s Morning Herald, an Australian health official allegedly managed to steal US$ 16 million (€ 12 million) that were intended for charities. He has been arrested this year in Brisbane. Authorities gave statement that Mr Hohepa Morehu-Barlow (also known as Joel Barlow) managed to misappropriate this amount over three years from Queensland Health, for which he worked as a finance manager. He allegedly used the money to fund a lavish lifestyle, occasionally even passing himself off as a “Prince of Tahiti” in social circles. The local police said that this was one of the most significant fraud cases in the history of Australia.

### Australian researchers estimate trends in global prevalence of diabetes

Three researchers from Baker IDI Heart & Diabetes Institute in Melbourne used studies from 91 countries to estimate national prevalences of diabetes for all 216 countries for the years 2010 and 2030. They estimated the world prevalence of diabetes among adults (aged 20-79 years) to be 6.4% in 2010, affecting 285 million adults, and that it would be expected to increase to 7.7% (and 439 million adults) by 2030. Between 2010 and 2030, they expect a 69% increase in numbers of adults with diabetes in developing countries and a 20% increase in developed countries.

## China

### China's influence rapidly growing in Africa

The Wall Street Journal reported on China expanding its economic and political ties with countries across Africa, resulting in a rapid rise in influence. They cite US officials saying how African governments find favor with China's “state-led capitalism” path of development, which gives Chinese firms an advantage over US competitors. Mr Robert D. Hormats, the US State Department's Under Secretary for Economic Affairs, said that this was “...part of a broad notion that China's economic model is successful and can be used elsewhere”. China is now the continent's largest trading partner, with its trade with Africa reaching US$ 114 billion (€ 88 billion), which is up from US$ 10 billion (€ 8 billion) in 2000 and US$ 1 billion (€ 0.8 billion)in 1980, according to China's State Council. Mr Mthuli Ncube, Chief Economist at the African Development Bank Group, estimated that “...Chinese firms accounted for 40% of the corporate contracts signed last year, to 2% for US firms”. While the US often sends aid money to non-governmental groups, China mostly provides aid through government entities in consultation with leaders about what their priorities are.

### China raises poverty bar to US$ 1 a day, up from 32 cents

China has taken the step of raising its poverty line to the UN’s recommended standard of US$ 1 (€ 0.8) a day. Before this move, the poverty line in China was set at 1196 yuan per year per resident, which was less than 32 US cents a day. For China, this means that the number of Chinese qualified as “poor” will suddenly increase by over 100 million – to about 128 million. All of those people will now become entitled to the government’s poverty alleviation program. This increase reflects a change in the financial might of China and their attitudes towards the poor. Before China’s rise as an economical power, its priorities in regards to the poor were restricted to adequately feeding and clothing the population. But as GDP per capita has risen (from about 858 yuan in 1985, when China started its economic reforms, to 30 000 yuan in 2010), the ability of China to support its massive population has increased. This rise in GDP, as the China Daily reported, means that China now has the means to broaden its welfare program beyond the basic needs. Improving the lives of its poorest citizens is now the key aim of the Chinese poverty alleviation program.

### Global Fund withholds nearly US$ 100 million intended for China’s AIDS fight

The Global Fund to Fight AIDS, Tuberculosis and Malaria will discontinue funding support for HIV/AIDS programs in China. The Global Fund’s spokesman Jon Liden said in the Geneva that the organization would keep about US$ 95 million (€ 73 million) from the total of US$ 270 million (€ 208 million) in grant money that was originally intended for China. This move was decided after a considerable pressure from both donors and non-governmental organizations to either fully stop or substantially reduce funding to China. Some of them quoted possible concerns over management of disbursed funds as part of the reason, while others wanted to see the increase in funding in low-income countries where AIDS is relatively much bigger problem, but which have far fewer resources to tackle it. Apparently, this Global Fund’s decision was not one-sided; it was made in consultation with Chinese officials.

### China vaccinates millions in western regions to contain polio outbreak

Chinese government moved to vaccinate more than 9 million people in western regions against polio amid an outbreak that left 17 paralyzed and one dead. China had been polio-free for eleven years before the new cases were reported in Xinjiang province. This outbreak exposed gaps in immunization coverage in this remote region, where access to health services is rather low. According to the WHO, the polio strain was probably introduced from Pakistan, which is bordering Xinjiang province and is also one of the four countries where polio remains endemic – along with India, Afghanistan and Nigeria.

### Chinese government and The Gates Foundation to collaborate in emerging technologies research

China's Ministry of Science and Technology and The Gates Foundation signed a memorandum of understanding under which they plan to invest together in research and development of new technologies that could improve global health and agriculture. The project, worth US$ 300 million (€ 232 million), will see every dollar provided from The Gates Foundation in support to selected China-grown products and technologies matched with US$ 2 (€ 1.6) as grant money from the Chinese Ministry. The list of considered technologies will likely be dominated by research in human and animal vaccines, diagnostics for tuberculosis and other diseases, varieties of resistant rice and other crops and more productive livestock.

## Europe

### Measles outbreaks reported across Europe

Recent reports from the Centre of Disease Control and Prevention confirmed that the incidence of measles was on the rise in Europe. From 2003, outbreaks of measles were steadily declining within the region, and the WHO target of measles eradication in Europe by 2010 seemed realistic. However, since 2009 rates of measles outbreaks have been increasing dramatically – with 36 of the 53 European member countries reporting outbreaks and more than 30 000 cases identified in 2010 alone. This trend has continued into 2011 and by November 2011 more than 26 000 measles cases had been confirmed. This figure has increased from less than 10 000 cases per year throughout 2007-2009. The key reason behind these figures is declining demand for vaccines and increased interest in the safety of the measles vaccine. In 2011, about half of cases of measles were in children under 15 years old with at least 45% of them unvaccinated. France has reported the greatest number of cases, about half of the entire burden in Europe in 2011. Largely, the answer to meeting the new target to eradicate measles is increase vaccination rates to maintain immunisation coverage to over 95% consistently across Europe.

### Drug-resistant tuberculosis rapidly spreading in Europe

The World Health Organization announced in September that “...drug-resistant forms of tuberculosis (TB) are spreading at an alarming rate in Europe”. TB is currently a worldwide pandemic that kills around 1.7 million people a year. Cases of multidrug-resistant (MDR-TB) and extensively drug-resistant TB (XDR-TB) — where the infections are resistant to first-line and then second-line antibiotic treatments — are spreading fast, with about 440 000 new patients every year globally. Half of the 30 countries with the highest burden of MDR-TB are in the WHO’s European region. More than 80 000 MDR-TB new cases occur in the European region each year, which is nearly a fifth of the world’s total. Officially reported cases of XDR-TB increased 6-fold between 2008 and 2009, with the rates highest in Eastern Europe and Central Asia. Treatment regimes for MDR-TB and XDR-TB can stretch into two or more years, costing up to US$ 16 000 (€ 12 000) in drugs alone and up to US$ 300 000 (€ 232 000) per patient in isolation hospital care. The risk of death from straightforward TB is about 7%, but it rises to nearly 50% among patients with drug-resistant forms.

### EU will discontinue aid to 19 middle-income countries from 2014

Economic crisis that grasped European continent will have consequences for the ability of the EU to aid other countries – especially given that some of them are already surpassing large EU economies. EU officials have decided that China and another 18 middle-income countries will no longer qualify to be the recipients of the European Commission's Development Cooperation instrument. Under the European Commission's new principle of “differentiation”, 19 middle-income countries whose GDP is now greater than 1% of global GDP will no longer be able to receive bilateral grant aid. Instead, they will benefit from possible new forms of partnership, which are being agreed. The other 18 countries on the list include Argentina, Brazil, Chile, Colombia, Costa Rica, Ecuador, Kazakhstan, India, Indonesia, Iran, Malaysia, Maldives, Mexico, Panama, Peru, Thailand, Uruguay and Venezuela.

### Dutch laboratory concerns health officials by mutating killer virus

Several high-level health officials from different countries expressed vigilance and concern after a Dutch laboratory managed to develop a mutant version of the deadly bird flu virus that is – for the first time – contagious among humans. A research team from Holland announced in September that it had created a mutant version of the H5N1 bird flu virus that could be spread among mammals. Later in the year, it has been revealed that the US government has paid scientists to try to understand how the deadly bird flu virus might mutate to become a bigger threat. Two laboratories – one in the US and one in Netherlands – apparently succeeded in understanding this. However, US federal officials then took the unprecedented step of asking the scientists who succeeded to restrain from publishing the details of their work.

### Russia plans aid for HIV-troubled eastern Europe and central Asia

According to Financial Times, Russia plans to offer aid to fight HIV in eastern European countries and central Asia. This move, which is seen by some analysts as Russia’s latest effort to restore some of its political influence among the former Soviet Union affiliates, should offer additional funding to tackle HIV in a region with the fastest continued HIV growth anywhere in the world. The infections have tripled over the past ten years and it is estimated that some 1.4 million people are now affected. Some journalists in recipient countries expressed concern that, with this welcome aid funding, Russia could also export its restrictive policies on HIV prevention methods.

## India

### National Rural Health Mission “a minor success”

An official review of the Indian Government's ambitious National Rural Health Mission described it as a “minor success”, adding that the results have been heartening compared to past experience in public health programmes. Deployment of human resources in the health sector has improved modestly, even though huge gaps still existed before the primary health care system could be declared to be running optimally, the report concluded. The review panel recommended that if the gains from this programme were to be consolidated, a renewed commitment for at least another seven years would be essential. The report called for almost quadrupling the per capita allocation for the health sector during the 12th five-year-plan period.

### India makes steady progress in infant mortality reduction

The results of the Coverage Evaluation Survey conducted by UNICEF in India showed that infant mortality rate in this large country has come down from about 58 per 1000 live births in 2005 to about 50 per 1000 in 2009. In addition, maternal mortality ratio has also come down: it stood at 254 per 100 000 live births for the 3-year rolling period between 2004 and 2006, but it decreased to 212 per 10 000 live births between 2007 and 2009. The immunization programme is being successfully rolled out throughout most of the country. It is estimated that up to 61% of children aged between 1 and 2 years in India are now fully immunized against 6 major vaccine preventable diseases. In December this year, pentavalent vaccination will also finally begin in several Indian states.

### Twenty three children allegedly contracted HIV at a hospital in India

Large sections of the India media reported in September that at least 23 children in western India were reported to have tested positive for HIV/AIDS after receiving blood transfusions at a state-run hospital in Junagadh, Gujarat. The children – all under the age of 10 years – were among around 100 children with thalassemia, who had been receiving free blood transfusions at the hospital since January 2011. The India Today, India's leading news magazine, reported that the hospital did not have facility for advance screening for HIV in blood. There are fears that many more children could have been infected. However, the hospital authorities have refused to accept any responsibility. They say that the patients had arranged for the blood from blood banks themselves and the hospital had only handled the transfusions. The state government has instituted an investigation.

### India gets closer to Polio Eradication

It has been over 300 days since last and only confirmed case of paralysis by wild polio virus in 2011. The case occurred in the eastern state of West Bengal in January 2011. Until the last year, India was still considered one of the polio endemic countries in the world. This apparent success in polio eradication is a result of a massive immunization drive launched by the Indian government and international partner agencies, which targeted high risk areas and introduced the new bivalent oral polio vaccine. The Indian government, however, remains cautious and plans to maintain high standards of polio surveillance.

### Japanese encephalitis kills hundreds in India

Japanese encephalitis (JE), a deadly viral disease caused by flavivirus and transmitted by Culex mosquitoes, has killed around 900 people across India, many of them children, according to data available until the end of November 2011. The state of Uttar Pradesh in northern India has been the worst hit so far, with more than 500 reported dead. The outbreak has spread to other states like Assam, West Bengal, Tamil Nadu and Haryana. The districts of Eastern Uttar Pradesh have had similar large scale JE outbreaks in the past, with the last one reported in 2005. The Indian health minister informed the Parliament that appropriate control measures – early case detection and proper case management, JE vaccination and health campaigns promoting cleanliness, sanitation and safe drinking water have been instituted.

## The Americas

### Latin America enjoys lowest poverty levels for 20 years

In November this year the Economic Commission for Latin America and the Caribbean (ECLAC) announced the lowest levels of poverty in Latin America for two decades. Poverty rates decreased from 48% in 1990 to 31% in 2010, which is a drop of 17 percentage points. Meanwhile, indigence rates – those living below the minimum subsistence level – dropped from 23% to 12% over the same time period. In 2011 poverty was predicted to fall one further percentage point, to 30.4%, meaning that at the end of 2011 ECLAC foresees a total of 174 million inhabitants living in Latin America in poverty. Indigence rates were set to rise by 0.5% percentage points to 12.8% in 2011, translating to 73 million inhabitants defined as living in extreme poverty or indigence. This was linked to expected increase in prices of living counteracting the forecasted increase in household income.

### Mysterious epidemic of deadly kidney disease spreads across Central America

According to BBC News, a mysterious epidemic is affecting the population of Central America. Reportedly, it has become the second most important cause of death among men in El Salvador. In Nicaragua, it's a bigger killer of men than HIV and diabetes added together. The epidemic extends beyond those two countries and it is prevalent along the Pacific coast of Central America, across six countries. Dr Victor Penchaszadeh, a clinical epidemiologist at Columbia University in the US and consultant to the Pan-American Health Organization on chronic diseases in Latin America, stated about this epidemic: “It is important that the chronic kidney disease (CKD) afflicting thousands of rural workers in Central America be recognized as what it is – a major epidemic with a tremendous population impact”. Reportedly, El Salvador's health minister recently called on the international community for help. Up to the quarter of the farming workers in the area seems to be suffering from the disease, which eventually kills them. Interestingly, most of those affected show no signs of high blood pressure or diabetes, which are the most common causes of CKD elsewhere in the world. Currently suspected risk factors that could be causing this kidney damage are the toxic chemicals – pesticides and herbicides – that are routinely used in agriculture in Latin America, but banned in the United States, Europe and Canada. However, the overuse of painkillers and alcohol abuse can also damage kidneys, and both are also prevalent among the affected population.

### United States grow increasingly concerned over biological weapons threat

Reporting on the Secretary of State Hillary Clinton’s visit to Geneva, Reuters wrote that the United States are now calling for closer international cooperation to prevent terrorist groups from developing or using biological weapons. In the era of exploding advances in genomic research of many living species, including potentially deadly viruses and bacteria, and widely commercially available technologies for genome manipulation, this threat is continuously growing. Ms Clinton was reported to have concluded: “Unfortunately, the ability of terrorists and other non-state actors to develop and use these weapons is growing. Therefore this must be a renewed focus of our efforts”,

### Brazil’s transition from aid recipient to an important aid donor

Brazil’s economy has just surpassed UK’s to become the 7th largest in the world. Although it is still an aid recipient, with a lot of inequity and poverty remaining to be tackled at home, Brazil is also growing a foreign aid programme of its own, which now amounts to nearly US$ 1 billion (€ 0.8 billion). However, aid itself is not the only contributor to Brazil’s growing international presence. Trade between Brazil and Africa has grown from US$ 5 billion (€ 3.9 billion) in 2003 to more than US$ 20 billion (€ 15.4 billion) in 2010 – and a third of the latter figure is generated through exchange with Nigeria alone. President Luiz Inácio Lula da Silva’s vision, highlighted in his bold “declaration of international relevance”, saw Brazil establishing 17 embassies in Africa, while Mr da Silva visited 23 African countries himself. Mr Marco Farani, the director of the Brazilian aid agency, said for The Guardian that his attitude to international aid strategy is very relaxed: “We don't have a strategy”, he stated proudly. He said that his preference was to respond to requests for support, rather than spending time on comprehensive strategic planning.

### Cuba launches the first vaccine for treatment of advanced lung cancer

According to Xinhua agency, Cuban medical authorities started selling the world's first therapeutic vaccine against lung cancer. The CimaVax-EGF vaccine comes as a result of more than two decades of research into diseases related to tobacco smoking. It has been developed by researchers at the Center of Molecular Immunology (CIM) in Havana. The active ingredient is the epidermal growth factor (EGF), a protein which is considered a biomarker of uncontrolled cancer and cell proliferation. The head researcher of the project, Dr Gisela Gonzalez, said that “...the drug could turn the cancer into a manageable, chronic disease by generating antibodies against the proteins which triggered the uncontrolled cell proliferation”. This immunogenic vaccine is indicated for patients with advanced lung cancer which do not show positive response to chemotherapy or radiotherapy. The vaccine cannot prevent the disease, but according to Dr Gonzalez, it “...improves significantly the status of the critically ill patients”. She added that researchers at the CIM planned to use the same principle in treating other cancers, such as prostate, uterus and breast cancers.

